# Molecular mechanisms and therapeutic potential of natural products and traditional Chinese medicine formulas in ADHD: a review of preclinical evidence

**DOI:** 10.3389/fphar.2026.1766222

**Published:** 2026-03-20

**Authors:** Han Deng, Bo Li, Yuze Mu, Weili Xia, Ning Weng

**Affiliations:** 1 Shandong Provincial Key Medical and Health Laboratory of Shandong Mental Health Center, Shandong University, Jinan, Shandong, China; 2 Department of Public Health, The First Affiliated Hospital of Shandong First Medical University, Jinan, Shandong, China; 3 Department of Chinese Medicine, Shandong Mental Health Center, Shandong University, Jinan, China

**Keywords:** attention-deficit/hyperactivity disorder, gut–brain axis, molecular mechanisms, natural products, neurotransmitters, synaptic plasticity, traditional Chinese medicine formulas

## Abstract

Attention-deficit/hyperactivity disorder (ADHD) is a prevalent neurodevelopmental condition marked by inattention, hyperactivity, and impulsivity. Although current pharmacotherapies are effective for many patients, their clinical utility is frequently limited by insufficient efficacy, adverse effects, and concerns regarding long-term tolerability or patient acceptance. Accordingly, increasing research attention has been directed toward the therapeutic potential of traditional Chinese medicine (TCM) formulas and other natural products, which are thought to offer multi-target mechanisms and holistic regulatory effects. This review provides a critical synthesis of recent preclinical studies, focusing on the molecular mechanisms by which specific TCM formulas and natural products ameliorate ADHD-like behaviors. We delineate these actions across several biological scales, including the modulation of catecholamine neurotransmission, enhancement of neurodevelopment and synaptic plasticity, attenuation of neuroinflammation, and regulation of the gut–brain axis. In addition, this review highlights current limitations, including the overreliance on single animal models and the insufficient integration of pharmacokinetic and translational data. Finally, we discuss future research directions and aim to provide new perspectives for the development of natural product-derived interventions in ADHD management.

## 1 Introduction

Attention-Deficit/Hyperactivity Disorder (ADHD) is a prevalent neurodevelopmental disorder characterized by inattention, hyperactivity, and impulsive behavior. Symptoms typically manifest in early childhood and may persist into adulthood (Koirala et al., 2024). The global prevalence among children is estimated to range from 5% to 10%, with a significantly higher diagnostic rate in males than in females (Mowlem et al., 2019). Current diagnostic criteria are based on the Diagnostic and Statistical Manual of Mental Disorders (DSM) and the International Classification of Diseases (ICD), which define ADHD as a chronic pattern of symptoms present in multiple settings and not better explained by other disorders (Drechsler et al., 2020). Standard treatment guidelines advocate for psychoeducation, behavioral interventions, and pharmacotherapy with either stimulant or non-stimulant medications as the primary approaches (Popit et al., 2024; Posner et al., 2020; Solmi et al., 2025). Although these drugs are effective for many patients, their use may be limited by incomplete response, adverse effects and concerns about long-term safety or acceptability among families (Faraone et al., 2015; Goez et al., 2007; Kotsopoulos and Spivak, 2001), highlighting the need for alternative therapeutic strategies.

**FIGURE 1:**
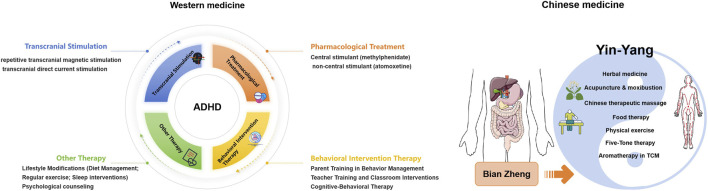
Comparison of the Western medicine and Chinese medicine treatment for ADHD. Western medicine primarily adopts evidence-based, symptom-targeted approach, focusing on improving core ADHD symptoms such as inattention, hyperactivity, and impulsivity. Its main treatment methods include: Pharmacological Treatment (This is a well-established and commonly used intervention), Behavioral Intervention Therapy, Transcranial Stimulation, Lifestyle Modifications and Psychological counseling. In Chinese medicine diagnosis, the focus is on syndrome differentiation known as “Bian Zheng”, emphasizing the balance of the body’s internal “Qi,” “Blood,” and “Zang-Fu Organs” (viscera). It views ADHD as a result of internal disharmonies, such as deficiency of Kidney essence, imbalance of Liver and Spleen, or accumulation of phlegm and heat. Common treatment methods include: Herbal Medicine, Acupuncture and Moxibustion, Chinese therapeutic massage, Food therapy, Physical exercise, Five-Tone therapy, Aromatherapy and other methods.

Traditional Chinese medicine (TCM) is a widely practiced system of medicine that posits human health is characterized by the dual forces of “yin” and “yang,” along with five organ systems. “Yin” represents calmness and tranquility, while “yang” symbolizes activity and impatience. The balance of these forces and organ systems is essential for physical and mental health, whereas imbalance can lead to conditions such as ADHD (Figure 1). Based on its clinical characteristics, ADHD is classified into the categories of “restlessness”, “deficiency and irritability” or “amnesia”. Its etiology primarily involves congenital deficiency, acquired improper care, trauma, illness, emotional disorders and other factors. Pathologically, it is primarily associated with dysfunction of the heart, liver, spleen, and kidney. Deficiency of both the heart and spleen leads to a loss of nourishment to the mind, resulting in inattention; exuberant liver yang can cause emotional fluctuations and impulsive behavior; liver and kidney yin deficiency often leads to insufficient essence and blood, resulting in hyperactivity; phlegm-heat disturbing the heart, arising from internal phlegm dampness, leads to restlessness and distractibility. Additionally, congenital insufficiency, such as kidney essence deficiency, may also be a contributing factor. In terms of treatment, TCM emphasizes syndrome differentiation to improve symptoms by regulating organ function and harmonizing qi and blood (Yuan H. et al., 2018). Depending on the source of the imbalance, various herbal formulations can be developed to address deficiencies and restore harmony (Liang et al., 2020).

TCM, which includes herbal medicine, acupuncture, dietary therapy, and physical exercise, is widely used for the treatment of various diseases, with herbal medicine being the most commonly applied modality, as illustrated in Figure 1 (Guo et al., 2024; Li W. et al., 2023). Modern neurobiology conceptualizes ADHD as a heterogeneous disorder involving dysregulation of catecholamine neurotransmission, alterations in cortical and subcortical development, disrupted synaptic plasticity and connectivity, and low-grade neuroinflammatory and metabolic changes (Posner et al., 2020). Preclinical studies in animal models have demonstrated that botanical formulations and their bioactive metabolites regulate dopamine and norepinephrine pathways, enhance neuroprotective signals, improve synaptic structure, reduce inflammatory cascades and oxidative stress, and modulate the gut–brain axis (Liang et al., 2020; Ni et al., 2015; Yuan H. et al., 2018). These findings suggest that such multi-target mechanisms may effectively address the complex symptomatology of ADHD. However, the existing evidence remains fragmented across disparate metabolites and models, and a systematic synthesis of these mechanistic insights is lacking. Therefore, this review aims to summarize preclinical studies of TCM formulas, single botanical drugs, and their active metabolites in the treatment of ADHD. By elucidating their complex molecular mechanisms of action, this review aims to provide a rigorous scientific basis for their future translation into clinical applications.

## 2 Literature search and methods

Although this manuscript was designed as a narrative rather than a systematic review, a structured literature search was undertaken to enhance completeness and transparency. We searched PubMed and Web of Science from database inception to February 2026. We used the following keywords to search for relevant articles (“Attention-Deficit/Hyperactivity Disorder” OR “ADHD” OR “hyperactivity”) AND (“botanical drug” OR “herbal medicine” OR “herbal formula” OR “natural product” OR “metabolite”). Articles were restricted to those with full text available in English. The inclusion criteria focused on preclinical studies (*in vivo* and *in vitro*) investigating the modulation of ADHD-related pathways by TCM formulas, botanical drugs, or their isolated metabolites. We excluded studies not directly related to ADHD pathophysiology; conference abstracts, reviews, news articles, and non-research commentaries; and studies with incomplete data or those where full text was unavailable.

**FIGURE 2:**
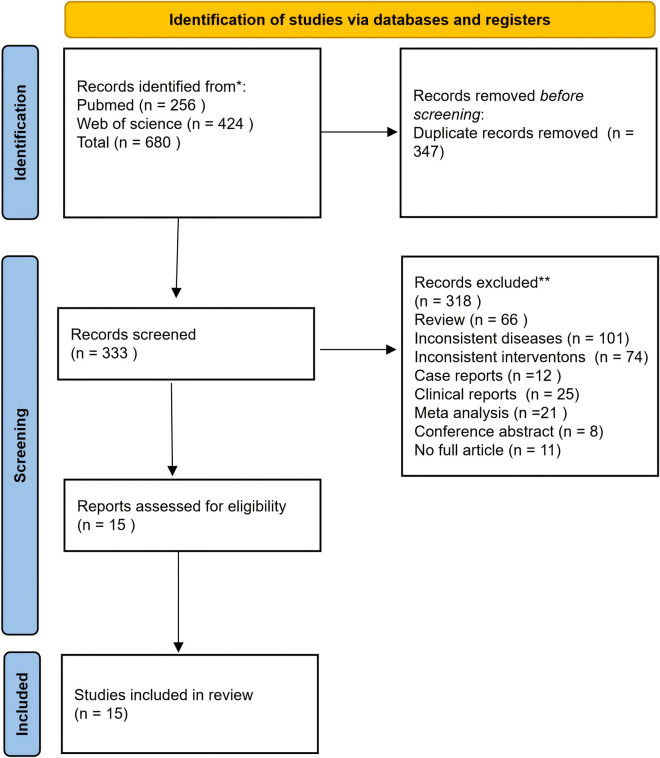
The PRISMA flowchart.

To ensure taxonomic and pharmacological rigor, studies were further screened for the inclusion of essential experimental details, such as dose ranges, specific models used, and chemical characterization of the material under investigation. The selection process and study flow are detailed in the PRISMA flowchart shown in Figure 2.

## 3 Molecular mechanisms of botanical interventions in ADHD

Preclinical evidence indicates that TCM formulas, botanical drugs, and their active metabolites alleviate ADHD symptomatology through pleiotropic mechanisms, including the modulation of neurotransmitter systems, enhancement of neurodevelopment and synaptic plasticity, regulation of neuroinflammation, and modulation of the gut–brain axis (Table 1).

**TABLE 1:** Pharmacological mechanisms of natural products and TCM formulas for the treatment of ADHD.

Natural products/TCM formulas	Constituents/source	Experimental model	Dosage	Main target	Main signaling pathways	Efficacy	Ref.
Jingning Granules	*Pseudostellaria heterophylla* (Miq.) Pax. (Taizishen) *Rehmannia glutinosa* (Gaertn.) Libosch. ex DC*.* (Shudihuang), *Lycium barbarum* L. (Gouqizi), *Schisandra chinensis* (Turcz.) Baill. (Wuweizi), *Polygala tenuifolia* Willd. (Yuanzhi) *Acorus verus* (L.) Raf. (Shichangpu), and *Poria cocos* (Schw.) Wolf (Fuling)	SHR	(1) Low/medium/high dose: 5.785, 11.57, and 23.14 g/kg (2) 10.14 g/kg	(1) the number of neurons in the hippocampal CA1 area ↑; D1R and D2R in the striatum and hippocampus↑; cAMP↓; Ca2+↓; CaM↓; CaMKII ↑ (2) kynurenic/quinolinic acid ratio↑; The abundance and diversity of intestinal microorganisms, the beneficial bacteria in the intestine ↑	(1) cAMP/PKA pathway Ca2+/CaM-CaMKII pathway (2) tryptophan metabolic pathway; microbiota-gut-brain axis	(1) Promote the production of neurotransmitters (DA) (2) Restore the balance of the intestinal microbiota and correct the dysfunction of tryptophan metabolism through the gut-brain axis	Ding et al. (2022), Yang et al. (2026)
An Shen Ding Zhi Ling	*Bupleurum chinense* DC. (Chaihu), *Scutellaria baicalensis* Georgi (Huangqin), *Forsythia suspensa* (Thunb.) Vahl. (Lianqiao), *Polygala tenuifolia* Willd. (Yuanzhi)*, Acorus verus* (L.) Raf. (Shichangpu), *Bambusa textilis* McClurei (Tianzhuhuang) *Curcuma longa* L. (Yujin), *Senna tora* (L.) Roxb. (Juemingzi), *Uncaria rhynchophylla* (Miq.) Miq. (Gouteng), *Angelica sinensis* (Oliv.) Diels (Danggui), *Alpinia oxyphylla* Miq. (Yizhiren) and *Rehmannia glutinosa* (Gaertn.) Libosch. ex DC. (Shengdihuang)	SHR	(1) 27.4 g/kg (2) 21.25 g/kg	(1) BDNF, TrkB, p75, JNK1, and NF-κB in the PFC and hippocampus↑ (2) IL-1β, IL-4, IL-6, TNF-α and MCP-1↓; IL-10↑; microglia, astrocytes and mast cells in the PFC and hippocampus; p-p38 MAPK, p-JNK, p-IκBα and p-p65 NF-κB↓	(1) BDNF/TrkB pathway BDNF/p75/JNK1/NF-κB pathway (2) MAPK signaling pathway; NF-κB signaling pathway	(1) Increase DA concentration in the synaptic cleft (2) Inhibit inflammatory responses	Song et al. (2020), Yaqun et al. (2022)
Long Mu Qing Xin Mixture	*Rehmannia glutinosa* (Gaertn.) Libosch. ex DC*.* (Shudihuang) *Paeonia lactiflora* Pall. (Baishao), *Os Draconis*. (Longgu), *Ostrea gigas* Thunberg (Muli), *Uncaria rhynchophylla* (Miq.) Miq. (Gouteng)	SHR	Low/medium/high dose:5.28 mL/kg/d; 10.56 mL/kg/d; 21.12 mL/kg/d	DA, NE, AC, cAMP, PKA, p-CREB, BDNF, D1, Gαs, Gαolf ↑	D1/cAMP/PKA- CREB signaling pathway	Increase catecholamine neurotransmitters	Li X. et al. (2024)
Xiaoer Huanglong Pellets	*Rehmannia glutinosa* (Gaertn.) Libosch. ex DC*.* (Shudihuang) *Paeonia lactiflora* Pall. (Baishao), *Ophiopogon japonicus (*Thunb.) Ker Gawl. (Maidong), *Anemarrhena asphodeloides* Bunge. (Zhimu), S*chisandra chinensis (Turcz.)* Bail. (Wuweizi), *Codonopsis pilosula* (Franch.) Nannf. (Dangshen), and *Acorus verus* (L.) Raf. (Shichangpu)	SHR	5.76 g/kg (WR_HL), 7.20 g/kg (CR_HL), 7.20 g/kg (JCR_HL)	BDNF, TH, DAT, and TPH2 ↑ Claudin-1, occludin, and ZO-1 ↑ TNF-α, IL-6 and IL-1β ↓ The abundance and diversity of intestinal microorganisms, the beneficial bacteria in the intestine ↑ Glu, His, Tyr, Trp, Ach, 5-HT, DA, NE, SCFAs ↑	microbiota-gut-brain axis	Optimize the bacterial community structure, restore the barrier function, regulate amino acid metabolism and SCFAs, ultimately improving neuroinflammation and neurotransmitter disorders	You et al. (2025)
Dimu Ningshen	*Rehmannia glutinosa* (Gaertn.) Libosch. ex DC. (Shudihuang), *Lycium barbarum* L. (Gouqizi), *Ligustrum lucidum* W.T.Aiton (Nv Zhenzi) *Cornus officinalis* Siebold & Zucc. (Shanzhuyu), *Schisandra chinensis* (Turcz.) Baill. (Wuweizi) *Dioscorea polystachya* Turcz. (Shanyao), *Anemarrhena asphodeloides* Bunge. (Zhimu), *Scrophularia ningpoensis* Hemsl. (Xuanshen), *Glycyrrhiza glabra* L. (Gancao), *Os Draconis*. (Longgu), *Ostrea gigas* Thunberg (Muli)	SHR	4.05 mg/kg	ALT, TBA, LDH-L, Cre, TG (No change); Ruminococcaceae_NK4A214_group, Ruminococcus_2, Eubacterium_nodatum_group ↑; peripheral monoamine neurotransmitter precursors ↑; peripheral fatty acid amides ↓	. Microbiota-gut-brain axis	Improve the structural composition of the gut microbiota and, at the same time, increase the circulating levels of peripheral polyamine neurotransmitter precursors (such as phenylalanine) and decrease the circulating levels of peripheral fatty acid amides (such as olemides)	Tang et al. (2022)
Sansoninto	*Ziziphus jujuba* Mill.var.*spinosa (Bunge) Hu ex H.F.Chou*, *Poria cocos* (Schw.) Wolf (Fuling), *Glycyrrhiza glabra* L. (Gancao), *Anemarrhena asphodeloides* Bunge. (Zhimu)	Social isolation mice	800 and 2400 mg/kg	Egr-1 ↑	Egr-1 signaling pathway	Promote neural synaptic plasticity	Fujiwara et al. (2018)
Rehmanniae Radix Preparata	the root tuber of the plant Rehmannia glutinosa	SHR	2.4 g/kg	The structure of the hippocampal region ↑; The number of hippocampal neurons in the CA1 region ↑; The number of TUNEL-positive cells ↓; Synaptic plasticity of the hippocampal DG ↑; neurogenesis in the hippocampus ↑; TrkB, Cdk5, FGFR1 ↑	BDNF/TrkB pathway; FGF/FGFR pathway	Reduce neuronal loss and increased the number of hippocampal stem cells, and promoted synaptic plasticity	Sun et al. (2024)
Baicalin	*Scutellaria baicalensis* Georgi	SHR	(1) Low/medium/high dose:50 mg/kg; 100 mg/kg; 150 mg/kg (2) 150 mg/kg	(1) TH, SNAP25, VMAT2, and syntaxin 1a ↑; DA ↑ (2) 8-OHdG ↓; CAT, GSH, and T-AOC ↑; Nrf2, Keap-1, and HO-1 ↑; TNF-α ↓; DAT ↓; VMAT2 ↑; p-NF-κB P65 ↓; HSC70 ↑	(1) SNAP25/syntaxin 1a (2) Nrf2/Keap-1/HO-1 pathway	(1) Promote DA synthesis and release (2) Activation of the Nrf2/Keap-1/HO-1 signaling pathway restored the balance of DAT and VMAT2 transport, and inhibited inflammatory responses, as well as reshaping the dopamine homeostasis in the brain	Ding et al. (2025), Zhou et al. (2019)
Saikosaponin A	*Bupleurum chinense* DC	SHR	25 and 50 mg/kg	DA ↑; DAT ↓; BDNF ↑	_	Increase neurotransmitters	Jichao et al. (2017)
Gastrodin	*Gastrodia elata* Blume	bisphenol A	30 and 60 mg/kg	GSH, MAO activity, and DA ↑; GFAP-positive cells in the cerebral cortex and hippocampus ↓	_	Increase neurotransmitters, Inhibition of lipid peroxidation and inhibition of astrocyte activation	Saifi et al. (2025)
Catalpol	*Rehmannia glutinosa* (Gaertn.) Libosch. ex DC.	SHR	50 mg/kg	MBP and NeuN expression in PFC and striatum ↑; BDNF ↑; Cdk5/p35 activity ↑; FGF21 and FGFR1 ↑	Cdk5/p35 FGF/FGFR pathway	Inhibit lipid peroxidation, reduce inflammatory response	Yuan et al. (2019)
Rhynchophylline	*Uncaria rhynchophylla* (Miq.) Miq	*In vivo*: Male DAT-KO Mice *In vitro*: LPS-Treated Microglia and Astrocytes	30 mg/kg; 20 mM	GFAP and CD11b-positive cells ↓ TNF-α, IL-1β, iNOS, and COX-2 in microglia and astrocytes ↓ TNF-α and IL-1β in cortical homogenates ↓	_	Promote neural development	Li J. et al. (2024)

### 3.1 Neurotransmitter system

**FIGURE 3:**
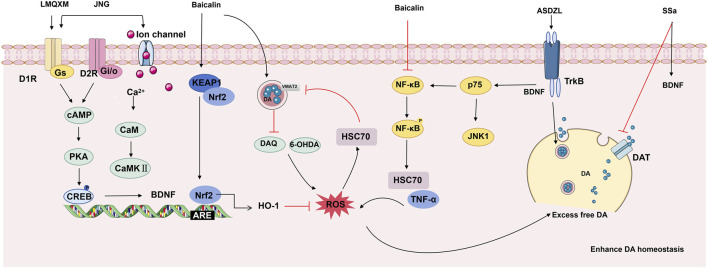
TCM ameliorates ADHD by promoting neurotransmitters. TCM (formulas and their isolated metabolites) alleviates ADHD symptoms by modulating neurotransmitter levels in the brain. LMQXM improves hyperactivity and learning/memory deficits in SHR by activating the DRD1/cAMP/PKA-CREB signaling pathway and increasing catecholamine neurotransmitter levels. JNG coordinates the balance between D1-and D2-like receptors, as well as their downstream cAMP/PKA and Ca^2+^/CaM/CaMKII signaling cascades, ultimately improving impulsivity, learning, and cognitive functions. ASDZL attenuates hyperactivity in SHR by regulating the balance between the BDNF/TrkB pathway (promoting vesicle cycling) and the BDNF/p75/JNK1/NF-κB pathway (inhibiting vesicle cycling) within synaptic compartments, thereby increasing dopamine concentration in the synaptic cleft. By activating the Nrf2/Keap-1/HO-1 pathway, baicalin significantly reduces oxidative stress and neuroinflammation, thereby restoring the DAT-VMAT2 transport equilibrium and enhancing DA stability. SSa promotes brain BDNF expression and simultaneously inhibits DAT-mediated dopamine reuptake, raising synaptic dopamine concentrations and alleviating ADHD-like symptoms. (Abbreviations: cAMP, cyclic adenosine monophosphate; PKA, protein kinase A; CREB, cAMP response element-binding protein; CaMKII, Ca^2+^/calmodulin-dependent protein kinase II; NF-κB, nuclear factor kappa-light-chain-enhancer of activated B cells; JNK1, c-Jun N-terminal kinase 1; LMQXM, Long Mu Qing Xin Mixture; JNG, Jingning Granules; ASDZL, An Shen Ding Zhi Ling; SSA, Saikosaponin A.)

Imbalances in catecholamine neurotransmitters, particularly dopamine (DA) and norepinephrine (NE), play a crucial role in the pathophysiology of ADHD (Li X. et al., 2024). The hydroxylation of DA is essential for NE production, and both neurotransmitters are critical for executive function through their neuromodulatory effects on frontal-striato-cerebellar circuits (Del Campo et al., 2011). Reduced concentrations of DA and NE have been observed in the serum of children with ADHD, potentially reflecting a disrupted central catecholaminergic state (Xiong et al., 2021). Rectifying this abnormal catecholaminergic neurotransmission is a crucial aim in treating ADHD. Spontaneously hypertensive rats (SHR) serve as a well-validated animal model for ADHD research, exhibiting key features of ADHD at 4–10 weeks of age, including inattention, impulsivity, and hyperactivity (Regan et al., 2022). A large number of experiments have confirmed that various botanical metabolites have demonstrated significant modulatory effects in ADHD models (Figure 3). For instance, Saikosaponin A (SSa), the primary triterpene saponin isolated from *Bupleurum chinense* DC., has been shown to increase DA concentrations and brain-derived neurotrophic factor (BDNF) levels in the prefrontal cortex (PFC) and striatum of SHR (Jichao et al., 2017). The DA transporter (DAT) facilitates extracellular DA reuptake, ultimately leading to DA degradation (German et al., 2015; Omiatek et al., 2013). The study has confirmed that elevated DA may be associated with reduced DAT (Jichao et al., 2017). Baicalin is a flavonoid purified from the plant *Scutellaria baicalensis* Georgi. *Zhou* et al. demonstrated that baicalin targeted the striatum and increased DA levels only in the striatum (Zhou et al., 2019). Furthermore, baicalin was found to enhance DA homeostasis by restoring DAT-vesicular monoamine transporter 2 (VMAT2) transport balance and reducing oxidative stress and inflammation by activating the nuclear factor erythroid 2-related factor (Nrf2)/kelch-like ECH-associated protein 1 (Keap-1)/heme oxygenase-1 (HO-1) pathway (Ding et al., 2025).

Beyond isolated metabolites, the complex pharmacological mechanisms of multi-herb TCM formulas have garnered considerable research attention. Jingning granules (JNG), a potent TCM formula developed from years of clinical expertise in treating ADHD, has been recognized with a national invention patent (Patent No. ZL201510303541.6). JNG can effectively improve the dysfunction of dopaminergic neurons in SHR. The mechanism may be through reducing the excessive function of D1-like receptors and promoting cyclic adenosine monophosphate (cAMP)/protein kinase A (PKA) and Ca^2+^/calmodulin (CaM)-dependent protein kinase II (CaMKII) signaling pathway (Ding et al., 2022). An shen ding zhi ling (ASDZL), a TCM formula, has been extensively utilised for the treatment of ADHD within the province of Jiangsu in China. ASDZL has been shown to alleviate ADHD symptoms in SHR rats by modulating the balance between the BDNF/tropomyosin receptor kinase B (TrkB) (which promotes vesicle circulation) and BDNF/p75/C-Jun N-terminal kinases 1 (JNK1)/nuclear factor kappa B (NF-κB) signalling pathway (which inhibits vesicle circulation) within the PFC and the hippocampus synaptosome, consequently increasing DA concentration in the synaptic cleft (Yaqun et al., 2022). Additionally, Longmu Qingxin Mixture (LMQXM), a TCM formula specifically designed for ADHD, has been demonstrated to elevate brain DA and NE levels *via* the D1/cAMP/PKA-cAMP response element-binding protein (CREB) signaling pathway (Li X. et al., 2024; Li X. et al., 2023).

### 3.2 Neural development and plasticity

**FIGURE 4:**
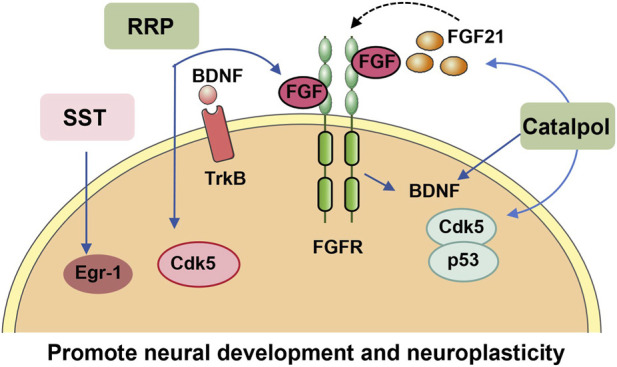
TCM ameliorates ADHD by promoting neurodevelopment and synaptic plasticity. TCM (formulas, single botanical drugs, and their metabolites) alleviates ADHD symptoms by enhancing neurodevelopment and structural synaptic plasticity in the brain. SST upregulates Egr-1 expression and enhances synaptic plasticity in the hippocampus. RRP reduces neuronal apoptosis, increases the hippocampal neural stem cell population, and promotes synaptic plasticity by regulating the BDNF/TrkB, Cdk5, and FGF/FGFR signaling pathways. Catalpol upregulates the expression of neurodevelopment-related proteins in the prefrontal cortex (including BDNF, Cdk5, p35, FGF21, and FGFR1) and improves hyperactivity, impulsivity, and spatial learning/memory in SHR, underscoring its critical role in neural maturation pathways. (Abbreviations: RRP, *Rehmanniae Radix Preparata*; FGF, fibroblast growth factor; SST, Suan Zao Ren Tang; TrkB, tropomyosin receptor kinase B; EGF, epidermal growth factor; FGFR, fibroblast growth factor receptor; Cdk5, cyclin-dependent kinase 5; Egr-1, early growth response protein 1; NRG-3, neuregulin-3.)

ADHD arises from a multifactorial etiology, and neuroimaging investigations have revealed that multiple critical brain regions in individuals with ADHD exhibit reduced volume or delayed maturation, as documented in prior research (Ming and Song, 2011; Song et al., 2016). Accumulating evidence has confirmed that restoring neuroplasticity in ADHD model rats effectively mitigates ADHD-related symptoms, with recent findings consistently supporting this therapeutic effect (Anderson et al., 2024; Marques et al., 2023; Sun et al., 2024). Neurogenesis persists throughout the mammalian lifespan, with the dentate gyrus (DG) of the hippocampus identified as its primary site (Ming and Song, 2011). In pediatric populations, the hippocampus is particularly notable, as it undergoes the most significant volume reductions and maturational delays compared to other brain regions (Hoogman et al., 2017) (Figure 4).

This link between hippocampal dysfunction and ADHD has directed attention to botanical drug interventions, with a data mining analysis identifying *Rehmanniae radix* preparata (RRP) as the most frequently utilized botanical drug remedy for ADHD treatment (Jia et al., 2023). Subsequent experimental validation confirmed that RRP exerts neuroprotective effects by reducing neuronal loss, expanding the population of hippocampal stem cells, and enhancing synaptic plasticity. The underlying mechanism may involve upregulation of the fibroblast growth factor (FGF)/fibroblast growth factor receptor (FGFR) signaling pathway (Sun et al., 2024). Beyond the hippocampus, abnormal neurodevelopment of prefrontal-striatal circuits is also recognized as a key contributor to ADHD pathogenesis. Studies have found that RRP may improve spontaneous and impulsive behaviors by up-regulating BDNF/TrkB and Neuregulin 3 (NRG3) expression in the prefrontal cortex and striatum, thereby improving neuronal growth and maturation in SHR (Yuan H. et al., 2018; Yuan H. X. et al., 2018). In addition, catalpol, the active metabolite of RRP, has been shown to be involved in the upregulation of several regulatory proteins in PFC development, such as BDNF, cyclin-dependent kinase 5 (Cdk5), p35, FGF21 and its receptor (FGFR) 1 (Yuan et al., 2019).

Regarding animal models of ADHD, early post-weaning social isolation (SI) in rodents has emerged as a viable approach, as it induces behavioral abnormalities analogous to those observed in human ADHD—including heightened aggressive responses, attention-deficit-like behaviors, and hyperactivity (Abu-Elfotuh et al., 2023; Fujiwara et al., 2018). SI stress in mice was recently found to cause not only impaired social competence and spatial attention, but also cognitive deficits in fear conditioning tests. Suan Zao Ren Tang (SST, also known as Sansoninto), a TCM formula historically used to treat insomnia, depression, and neuropathy in adults, has shown therapeutic potential. Daily administration of SST was found to alleviate SI-induced impairments in episodic and auditory fear memory, while also reversing the downregulation of early growth responsive gene-1 (Egr-1) expression in the hippocampus and cortex of SI-exposed animals (Fujiwara et al., 2018).

### 3.3 Inflammation and immune regulation

**FIGURE 5:**
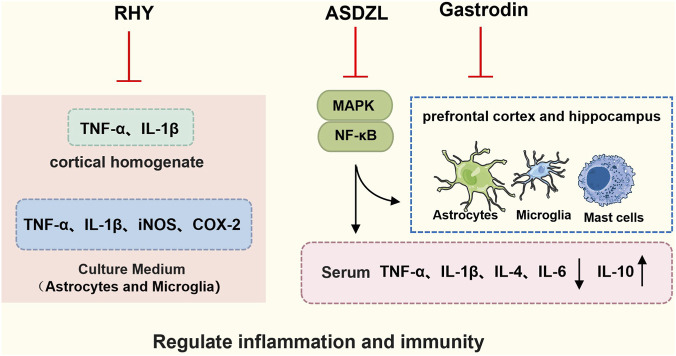
TCM ameliorates ADHD by regulating inflammation and immunity. TCM (formulas and their isolated metabolites) alleviates ADHD-like phenotypes by suppressing central inflammation and regulating neuroimmunity. Rhynchophylline mitigates hyperactivity and cognitive flexibility deficits in DAT-KO mice; *in vitro*, it inhibits the expression of pro-inflammatory mediators (TNF-α, IL-1β, iNOS, and COX-2) in microglia and astrocytes, while simultaneously suppressing TNF-α and IL-1β expression in cortical homogenates. ASDZL downregulates pro-inflammatory cytokines (IL-1β, IL-4, IL-6, TNF-α, and MCP-1) and upregulates the anti-inflammatory cytokine IL-10 in SHR. Concurrently, it reduces the morphological activation of microglia, astrocytes, and mast cells in the prefrontal cortex and hippocampus. Furthermore, ASDZL suppresses neuroinflammation by maintaining blood-brain barrier integrity and suppressing the MAPK and NF-κB signaling cascades. Gastrodin treatment reduces the density of GFAP-positive astrocytes in the cerebral cortex and hippocampus, effectively preventing bisphenol A-induced ADHD-like symptoms. (Abbreviations: TNF-α, tumor necrosis factor-alpha; IL-1β, interleukin-1 beta; IL-4, interleukin-4; IL-6, interleukin-6; IL-10, interleukin-10; iNOS, inducible nitric oxide synthase; COX-2, cyclooxygenase-2; ASDZL, An Shen Ding Zhi Ling; MAPK, mitogen-activated protein kinase; NF-κB, nuclear factor kappa-light-chain-enhancer of activated B cells.)

Neuroinflammation can lead to brain dysfunction and is evident in both neurological and psychiatric disorders (Bauer and Teixeira, 2019; Jeon et al., 2019). Research indicates that ADHD patients exhibit elevated concentrations of proinflammatory cytokines and reduced levels of anti-inflammatory cytokines as well as brain BDNF (Cortese et al., 2019; Darwish et al., 2019; Dunn et al., 2019; Elsadek et al., 2020). High concentrations of cytokines, chemokines, and oxidative stress markers have been identified in the serum of SHR (Kozłowska et al., 2019). Therefore, inhibiting neuroinflammation may represent an effective therapeutic strategy for ADHD (Figure 5).

ASDZL has been shown to significantly increase DA levels in SHR in previous studies, while recent studies have found that ASDZL effectively downregulates pro-inflammatory cytokines (IL-1β, IL-4, IL-6, TNF-α, MCP-1) while upregulating the anti-inflammatory cytokine IL-10. It concurrently reduces activation of microglia, astrocytes, and mast cells in the PFC and hippocampus. This anti-neuroinflammatory action likely involves suppression of the mitogen-activated protein kinase (MAPK) and NF-κB signaling pathways (Song et al., 2020). As a TCM formula, Ningdong granule (NDG) has been used to treat ADHD for several years in China. Clinical studies found that NDG improved ADHD symptoms after 8 weeks of drug treatment with fewer side effects compared to methylphenidate. NDG was safe and tolerable in children with ADHD as determined by blood, urine, and stool analysis and liver and kidney function monitoring for 8 weeks (Li et al., 2011). Subsequent preclinical studies extended these insights by identifying rhynchophylline, a major alkaloid constituent of NDG, as a key bioactive agent responsible for ameliorating hyperactivity and cognitive flexibility deficits in a DAT knockout (DAT-KO) mice model (Li J. et al., 2024). These effects were mechanistically linked to suppression of neuroinflammatory signaling pathways.

Gastrodin (GAS) is a bioactive pharmaceutical metabolite extracted from the dried roots of *Gastrodia elata* Blume. Network pharmacological analysis suggested that neuroactive ligand-receptor interaction, cholinergic synapses, and dopaminergic synapses may be the core pathways through which GAS exerts its therapeutic effects in ADHD (Song et al., 2022). In the latest study, *Saifi* et al. used Bisphenol A (BPA) to establish an ADHD rat model. After GAS treatment, it reduced lipid peroxidation, enhanced the monoamine oxidase (MAO) activity, and reduced the number of GFAP-positive cells in the cerebral cortex and hippocampus (Saifi et al., 2025).

### 3.4 Gut-brain axis interaction

The gut microbiota can directly or indirectly influence neurotransmitter production through the host’s biosynthetic pathways. Imbalance in this process can lead to disorders in neurotransmitters and neural development functions (Sittipo et al., 2022; Ullah et al., 2023). In recent years, the role of intestinal flora imbalance in the pathological mechanism of ADHD has gradually attracted attention (Gandhi et al., 2024; Han et al., 2025; Harigai et al., 2025; Leng et al., 2025; Lewis et al., 2025; Liu et al., 2025; Novau-Ferré et al., 2025; Steckler et al., 2024; Yin et al., 2024; You et al., 2025). Existing studies have confirmed that intestinal flora imbalance can contribute to ADHD-related pathological processes through multiple mechanisms. On the one hand, it can induce chronic intestinal inflammation, promote the accumulation of harmful metabolites, and directly damage the integrity of the intestinal epithelial barrier (Braniste et al., 2014; Kelly et al., 2015). On the other hand, neuroactive substances produced by microbial metabolism can cross the blood-brain barrier (BBB) through the blood circulation, interfere with the dynamic balance of neurotransmitters by regulating the synthesis, release and reuptake of neurotransmitters, and subsequently affect central nervous system function (Asano et al., 2012; Liu et al., 2025). More importantly, the bidirectional regulation mechanism of gut-brain axis plays a key role in this process (Checa-Ros et al., 2021; Gershon and Margolis, 2021).

**FIGURE 6:**
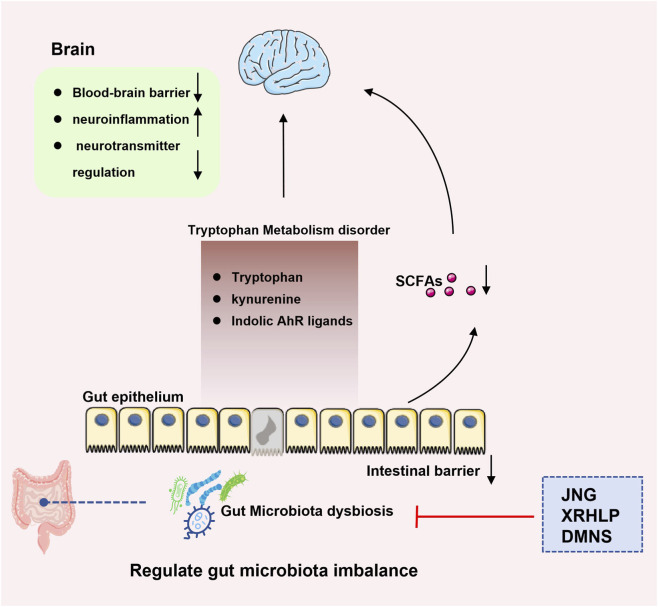
TCM ameliorates ADHD by regulating gut-brain axis. TCM formulas ameliorate ADHD-associated symptoms by comprehensively targeting the gut-brain interface. JNG restores intestinal flora balance and tryptophan metabolic homeostasis in ADHD models. XRHLP remodels gut microbiota composition and structure, corrects imbalances in amino acids, neurotransmitters, and SCFAs, attenuates neuroinflammation, and ultimately mediates beneficial alterations in the host’s peripheral microenvironment. DMNS alleviates ADHD-like behaviors by modulating the gut microbiota architecture and favorably altering peripheral circulating metabolite profiles. (Abbreviations: JNG, Jingning Granules; XRHLP, Xiaoer Huanglong Pellets; DMNS, Dimu Ningshen; SCFAs, short-chain fatty acids).

Several TCM formulas appear to act on ADHD through this gut-brain interface (Figure 6). A targeted-release formulation of Xiaoer Huanglong Pellets (XRHLP), designed for delivery to the stomach, intestine and colon, has been shown in SHR to restore microbial homeostasis, suppress excessive inflammatory cascades and repair both intestinal barrier and BBB structure, suggesting multi-level regulation of flora balance, inflammation, and barrier integrity (You et al., 2025). Notably, *Tang* et al. further validated the regulatory role of CM in the gut-brain axis of ADHD through a study on Dimu Ningshen (DMNS), an oral TCM formula. The study showed that DMNS could significantly modulate the gut microbiota structure of SHR, which reduced the abundance of ADHD-associated pathogenic microbiota such as Ruminococcaceae_NK4A214_group, Ruminococcus_2, Eubacterium_nodatum_group, and Christensenellaceae (which were highly abundant in the SHR group), and increased the proportion of beneficial bacteria including Bacteroidetes and Patescibacteria. Simultaneously, it increases circulating precursors of monoamine neurotransmitters and decreases fatty acid amides linked to ADHD, indicating coordinated modulation of microbiota-metabolite networks (Tang et al., 2022). These findings suggest that DMNS may exert its therapeutic effects in ADHD by regulating the interaction between gut microbiota and metabolites, thereby modulating the gut–brain axis. In addition, the commonly used drug JNG has been shown to increase the overall quantity and diversity of microorganisms, selectively promote the growth of beneficial intestinal bacteria, and increase the ratio of kynurenic/quinolinic acid. Such changes are generally considered to have neuroprotective effects (Yang et al., 2026).

## 4 Discussion

ADHD is a multifactorial neurodevelopmental disorder involving dysregulation of monoaminergic neurotransmission, impaired synaptic plasticity, neuroimmune activation, and disturbances in the gut–brain axis (Ding et al., 2025; Song et al., 2020; Sun et al., 2024; Yang et al., 2026). Although pharmacological interventions such as the stimulant methylphenidate (MPH) and the non-stimulant atomoxetine remain first-line treatments, their long-term tolerability, risk of adverse reactions, and incomplete symptom remission in a subset of patients underscore the need for complementary therapeutic strategies. In this review, botanical drugs and plant-derived bioactive metabolites have attracted increasing attention due to their multi-target regulatory characteristics. The studies summarized in this review suggest that botanical interventions may modulate dopaminergic and noradrenergic signaling, enhance plasticity-related pathways such as BDNF, attenuate microglial activation and inflammatory cascades, and restore gut microbiota balance. These findings collectively support the concept that botanical interventions exert systems-level modulation across interconnected pathological domains of ADHD.

Importantly, neurotransmission, synaptic plasticity, neuroinflammation, and the gut–brain axis are not independent domains but components of a dynamically interacting system. Pro-inflammatory cytokines can alter dopamine synthesis and transporter activity, thereby influencing executive function circuits in the prefrontal cortex (Yan et al., 2015). Conversely, dopaminergic signaling can modulate microglial activation states and immune tone (Yan et al., 2015). Gut microbiota-derived metabolites, including short-chain fatty acids and tryptophan metabolites, may further regulate both central neurotransmission and inflammatory responses through immune, endocrine, and neural pathways (Liu et al., 2024; Qiu et al., 2022). Within this integrative framework, the multi-target properties of botanical formulations appear theoretically aligned with the complex pathophysiology of ADHD. However, while conceptual coherence exists, direct experimental evidence demonstrating coordinated and causal interactions among these axes remains limited.

Despite encouraging mechanistic findings, the preclinical literature presents notable limitations. First, the majority of mechanistic investigations have been conducted using the SHR model. While SHR exhibit hyperactivity and certain catecholaminergic alterations, they represent only one ADHD-relevant phenotype. Very few botanical drugs have been evaluated in DAT-KO mice or neonatal 6-hydroxydopamine (6-OHDA) lesion models, which represent distinct pathophysiological mechanisms. The near-exclusive reliance on SHR restricts external validity and raises concerns about model-specific effects. Cross-model validation is essential to determine whether reported pharmacological actions generalize across ADHD subtypes. Second, pharmacokinetic profiling is rarely conducted. Consequently, brain exposure levels, dose–response relationships, and minimal effective concentrations often remain undefined. Third, the chemical standardization of plant extracts is frequently inadequate, with insufficient reporting on drug-to-extract ratios, metabolite quantification, or voucher specimen documentation. Fourth, the inclusion of positive control benchmarks is inconsistent, complicating the interpretation of pharmacodynamic outcomes. Collectively, these limitations constrain both reproducibility and the strength of translational inferences.

In addition to preclinical evidence, emerging clinical data also suggest that botanical treatment of ADHD may bring benefits (Ni et al., 2015; Qi et al., 2025). For example, a randomized controlled trial of Jingling Oral solution (Liaoning Oriyuan Pharmaceutical Co., LTD.) reported that the formulation improved total scores and hyperactivity/impulsivity subscale scores in children with ADHD, with good short-term safety (Qi et al., 2025). This study employed centralized randomization and a placebo-controlled parallel design, which reduces selection bias and enhances internal validity. However, despite these strengths, several methodological issues warrant careful consideration. Although the study was described as double-blind, botanical formulations inherently pose challenges in placebo matching due to their distinctive taste, color, and odor. The adequacy of blinding integrity was not formally tested, and no assessment of blinding success was reported, leaving potential performance and detection bias insufficiently addressed. Second, while the trial included a relatively larger sample compared with previous botanical drug studies, detailed justification of sample size calculation and power estimation for secondary outcomes was limited, raising questions about statistical robustness beyond primary endpoints. This represents a common limitation of current randomized controlled trials (RCTs) investigating botanical drugs for ADHD treatment. Furthermore, all available studies have been conducted exclusively in China, which constrains their generalizability due to regional and ethnic specificities. Consequently, while botanical drugs show promising therapeutic potential, high-quality, multicenter clinical trials involving diverse populations are urgently needed before their widespread application on a global scale can be justified.

Integrated traditional Chinese and Western medicine (ICWM) is an important approach in ADHD clinical practice in China (Wang and Zhang, 2017). In ICWM, Western medicine can quickly control the core symptoms through standardized drugs, while TCM can regulate the function of viscera, improve comorbidities (such as anxiety and sleep disorders) and reduce the side effects of Western medicine. For example, Huanglian Wen Dan Tang, a classical formula traditionally used to “clear heat and resolve phlegm” has been shown in pharmacological studies to modulate γ-aminobutyric acid type A (GABAA) receptor activity, suggesting a potential role in ameliorating insomnia (Klein-Schwartz, 2002; Li L. et al., 2024; Shi et al., 2025). Given that MPH treatment is frequently associated with sleep disturbances and irritability, it is theoretically plausible that such formulas may mitigate these adverse effects. However, it must be emphasized that these assumptions are primarily based on pharmacological inference rather than direct clinical evidence in ADHD populations. Although several clinical studies have explored ICWM approaches, standardized, evidence-based protocols have yet to be established. An early RCT by Lin et al. examined a 12-week course of combined MPH and Ningshen Oral Liquid in children with ADHD. The overall response rate in the combination group (83.76%) exceeded that of the Western medicine group (72.30%) and the TCM-only group (70.98%). Moreover, the incidence of adverse reactions was lower than in the MPH monotherapy group, although still higher than in the TCM-only group (Lin Yuin et al., 2007). These findings suggest potential benefits of integrative therapy, but fall short of defining a standardized, evidence-based ICWM regimen.

## 5 Conclusion and prospects

In recent years, the adjuvant treatment of ADHD with botanical drugs has attracted attention due to their favorable safety profiles and multi-target treatment characteristics. This comprehensive review systematically outlines the substantial preclinical progress achieved in elucidating the molecular mechanisms underlying botanical formulations in ADHD management. However, to maintain absolute scientific objectivity, the profound limitations of the current literature base must be explicitly acknowledged. These include the predominant use of a single animal model (SHR), substantial variability in botanical formulations, extraction methods, and dosing regimens across studies, as well as clinical investigations characterized by small sample sizes, geographic restriction to China, and a paucity of rigorous RCTs.

Future research should prioritize more in-depth mechanistic studies employing diverse experimental animal models to elucidate the complex “multi-component–multi-target–multi-pathway” network of actions underlying botanical interventions. At the preclinical level, cross-model validation using DAT-KO mice, neonatal 6-OHDA lesion models, and other ADHD-relevant models is essential to establish generalizability of findings. Additionally, pharmacokinetic profiling and standardized chemical characterization of botanical extracts should become mandatory components of preclinical investigations. At the clinical level, beyond increasing sample sizes and extending follow-up durations, more rigorous study designs are required, including the use of blinding and placebo controls, incorporation of objective biomarkers as adjunctive efficacy measures, and systematic assessment of quality of life and long-term functional outcomes.

Ultimately, advancing the standardization and individualization of integrative Chinese and Western medicine for ADHD is of great importance. Evidence-based treatment guidelines grounded in both pattern differentiation and disease diagnosis should be established to clarify the optimal timing, target populations, and regimens for combination therapy, with the aim of enhancing efficacy, reducing toxicity, and achieving both symptomatic relief and root-cause resolution, thereby offering more comprehensive and personalized treatment options for patients with ADHD worldwide.

## Glossary

ADHD: Attention-Deficit/Hyperactivity Disorder
DSM: Diagnostic and Statistical Manual of Mental Disorders
ICD: International Classification of Diseases
TCM: Traditional Chinese medicine
CM: Chinese medicine
DA: dopamine
NE: norepinephrine
SHR: Spontaneously hypertensive rats
SSa: Saikosaponin a
BDNF: brain-derived neurotrophic factor
PFC: prefrontal cortex
DAT: DA transporter
VMAT2: vesicular monoamine transporter 2
Nrf2: nuclear factor erythroid 2-related factor
Keap-1: kelch-like ECH-associated protein 1
HO-1: heme oxygenase-1
GAS: Gastrodin
BPA: Bisphenol A
MAO: monoamine oxidase
JNG: Jing ning granules
cAMP: cyclic adenosine monophosphate
PKA: protein kinase A
CaM: calmodulin
CaMKII: CaM-dependent protein kinase II
ASDZL: An shen ding zhi ling
TrkB: tropomyosin receptor kinase B
JNK1: C-Jun N-terminal kinases 1
NF-κB: nuclear factor kappa B
LMQXM: Longmu Qingxin Mixture
CREB: cAMP response element-binding protein
DG: dentate gyrus
FGF: fibroblast growth factor
FGFR: fibroblast growth factor receptor
SI: Social isolation
Cdk5: cyclin-dependent kinase 5
SST: Suan Zao Ren Tang
early growth responsive gene-1: early growth responsive gene-1
mitogen-activated protein kinase: mitogen-activated protein kinase
NDG: Ningdong granule
RHY: Rhynchophylline
BBB: blood-brain barrier
XRHLP: Xiaoer Huanglong Pellets
DMNS: Dimu Ningshen
ICWM: Integrated traditional Chinese and Western medicine
6-OHDA: 6-hydroxydopamine
RCT: Randomized controlled trial
